# Case Report: Hyperactive delirium with psychotic symptoms associated with severe hypothyroidism during pembrolizumab therapy. Complete remission after levothyroxine and no recurrence after pembrolizumab rechallenge

**DOI:** 10.3389/fpsyt.2026.1905725

**Published:** 2026-10-27

**Authors:** Eduardo Vieira Fregolente, Sabine Bühler, Jacob Lahr

**Affiliations:** 1 University Hospital of Old Age Psychiatry and Psychotherapy, University of Bern, Bern, Switzerland; 2 Prolindo Oncology and Hematology Practice, Bern, Switzerland

**Keywords:** case report, delirium, delusions, hypothyroidism, pembrolizumab, psychosis

## Abstract

Thyroid dysfunction is a common adverse event during pembrolizumab therapy, but severe hypothyroidism accompanied by acute neuropsychiatric symptoms is rare. We present the case of a 78-year-old woman with metastatic colorectal cancer who developed agitation, hallucinations, and confusion three months after initiation of Pembrolizumab. Evaluation revealed profound hypothyroidism (TSH 37.9 mU/L, free T3 <0.60 pmol/L and free T4 <0.5 pmol/L) and moderate hyponatremia without acute findings on neuroimaging. Levothyroxine therapy led to complete remission of neuropsychiatric symptoms, and pembrolizumab was safely restarted with close monitoring and no recurrence. This case underscores the importance of assessing thyroid function in acute psychiatric presentations during immune checkpoint inhibitor therapy and supports cautious rechallenge after stabilization. Severe hypothyroidism was considered a major contributor, although a multifactorial delirium, including the contribution of hyponatremia and the absence of CSF/EEG evaluation, cannot be excluded.

## 1 Introduction

Immune checkpoint inhibitors (ICIs) have transformed the treatment of several advanced malignancies but may cause immune-related adverse events (irAEs) affecting multiple organ systems. Endocrine toxicities are among the most frequent immune-related adverse events, with thyroid dysfunction being particularly common during programmed cell death protein 1 inhibitor therapy, including pembrolizumab (1, 2).

Most cases of immune checkpoint inhibitor-associated hypothyroidism are detected through routine laboratory monitoring or present with nonspecific systemic symptoms such as fatigue, cold intolerance, or weight gain (3). In contrast, severe hypothyroidism presenting predominantly with acute neuropsychiatric symptoms, including hyperactive delirium, hallucinations, and persecutory delusions, is rare and may be difficult to distinguish from other neurological, metabolic, or psychiatric causes (4).

We describe the case of a 78-year-old woman with metastatic colorectal cancer who developed hyperactive delirium with psychotic symptoms in the context of severe hypothyroidism during pembrolizumab therapy. The symptoms remitted completely after levothyroxine replacement, and pembrolizumab was subsequently restarted without recurrence under close laboratory monitoring.

## 2 Case description

A 78-year-old woman with metastatic colorectal cancer began palliative pembrolizumab on July 4, 2025. Baseline thyroid-stimulating hormone was normal at 1.99 mU/L. Relevant comorbidities included chronic kidney disease, KDIGO stage G2, and mild aortic stenosis. She had no known previous psychiatric history and had lived independently prior to the index presentation. Approximately three months after pembrolizumab initiation (after four administrations), the patient developed progressive confusion and functional cognitive decline over about three weeks, followed by marked agitation and psychotic symptoms with persecutory/poisoning delusions. On October 3, 2025, she was brought to the emergency department for severe disorganization and agitation; she was disoriented in all spheres with incoherent responses and a nonfocal neurologic examination.

During the subsequent medical hospitalization, the clinical picture fluctuated. She was intermittently more accessible but remained at times disoriented, motorically restless, and increasingly agitated, with prominent visual hallucinations, persecutory and poisoning delusions, ideas of external control, evening and nocturnal agitation, barricading behavior, and verbal and physical aggression toward staff.

The diagnosis of hyperactive delirium was made clinically, consistent with an acute/subacute disturbance of consciousness and cognition, disorientation, incoherent thought and speech, psychomotor agitation, hallucinations and delusions, sleep-wake disturbance, and a fluctuating course in the context of acute medical abnormalities.

On physical examination during the initial hospitalization, the patient was afebrile and hemodynamically stable, without focal neurological deficits. During psychiatric hospitalization, the thyroid was not enlarged or tender, no nodules were palpable, and no peripheral edema was present. Cardiovascular examination showed a regular normofrequent pulse and blood pressure within the normal range. Neurological examination showed normal strength and tone, lively symmetric reflexes, no pathological reflexes, and no ataxia.

Medical evaluation was notable for severe hypothyroidism. On September 8, 2025, during interval monitoring, TSH had already increased to 41.20 mU/L. On October 3, 2025, TSH was 37.90 mU/L, free T3 was below the assay lower limit (<0.60 pmol/L; reference 3.74-6.11), and free T4 was below the assay lower limit (<0.5 pmol/L; reference 11.5-19.6). The relatively modest TSH elevation despite very low free thyroid hormone levels was interpreted cautiously; a rapidly evolving ICI-related thyroiditis with incomplete TSH equilibration was considered possible, whereas a partial central component could not be fully excluded from the available data. Concomitant laboratory abnormalities included moderate hypoosmolar hyponatremia and mild hypokalemia. Sodium was 135 mmol/L on July 4, 2025, 130 mmol/L on September 8, 2025, 124–125 mmol/L on October 3, 2025, with serum osmolality 250 mOsm/kg, urine sodium 21 mmol/L, and urine osmolality 202 mOsm/kg. Sodium improved to 128 mmol/L on October 4, fluctuated between 126 and 131 mmol/L during the acute medical hospitalization, and was 130 mmol/L on October 10. After transfer to psychiatry, sodium increased from 129 mmol/L on October 15 to 132–133 mmol/L during October and normalized to 139 mmol/L on November 3. Potassium was 3.3 mmol/L on October 3 and normalized after supplementation. Corrected calcium was within the reference range. CRP was mildly elevated during the acute admission. Vitamin B12 was borderline low, folate was normal, and treponemal serology was negative during the psychiatric admission. Neuroimaging did not reveal an explanatory acute lesion: cranial CT showed a small punctate hyperdensity in the left parietal deep white matter interpreted as possible hemorrhage versus calcification, and subsequent MRI described an occipital cavernoma without acute hemorrhage. Available cortisol values did not support clinically overt adrenal insufficiency: cortisol was 269 nmol/L on July 4, 619 nmol/L on September 30, 492 nmol/L on October 4, and 516 nmol/L in January 2026. However, adrenocorticotropic hormone values, sampling details, and a comprehensive assessment of other pituitary axes were not available in the records reviewed. Thyroid antibody testing was not documented during the acute episode. Delayed follow-up testing in August 2026 showed negative TSH receptor antibodies (<0.8 U/L; reference <1.8), mildly elevated thyroperoxidase antibodies (44 IU/mL; reference <20), and negative thyroglobulin antibodies (20 IU/mL; reference <115). Because these measurements were obtained several months after onset, they could not reliably define antibody status during the acute thyroid dysfunction. Lumbar puncture and EEG were considered but were not performed, initially because of severe agitation and later because of rapid clinical improvement and unremarkable MRI findings.

The main differential diagnoses included immune checkpoint inhibitor-associated encephalitis, acute structural brain pathology, adrenal insufficiency or hypophysitis, metabolic encephalopathy related to hyponatremia, and a primary psychiatric disorder. However, the temporal association with pembrolizumab exposure, the profound biochemical hypothyroidism, the absence of previous psychiatric illness, and subsequent remission after thyroid hormone replacement supported severe hypothyroidism as the principal contributor to the hyperactive delirium with psychotic symptoms. However, causal attribution remains limited by the probable multifactorial nature of delirium and by the absence of CSF and EEG evaluation.

Pembrolizumab was paused, and Levothyroxine was initiated on October 3. During medical hospitalization, symptomatic treatment with sedating/antipsychotic agents was limited by medication refusal and insufficient clinical response; severe agitation required intravenous midazolam, and she was transferred to inpatient psychiatry (October 12). At psychiatric admission, she had an agitated delirium with psychotic features, including vivid visual hallucinations, delusions of poisoning and persecution, altered consciousness and few clear moments. She arrived on levothyroxine 50 micrograms/day; the dose was increased to 100 micrograms/day on October 23 and to 150 micrograms/day on November 6 because of persistent biochemical hypothyroidism. Clinically, psychotic symptoms and agitation regressed during the psychiatric admission, allowing discontinuation of benzodiazepines and tapering of risperidone. On October 21, she still reported persecutory ideas. By October 27, staff documented fewer psychotic statements and increasing independence. By October 31, she reported no longer feeling persecuted, describing the previous experience as similar to a nightmare. On November 6 and November 11, she was awake and clear in consciousness, calm, coherent, euthymic, and without hallucinations or delusional thought content. At discharge on November 12, 2025, she was fully oriented, coherent, without positive psychotic symptoms, independent in basic and complex activities of daily living. At follow-up in April 2026, she remains on levothyroxine 150 micrograms/day, and pembrolizumab has been restarted at 3-week intervals. The thyroid laboratory is monitored every 6 weeks. She lives independently, and tumor staging showed stable disease. The clinical course, key laboratory findings, and treatment timeline are summarized in **Table 1**.

**Table 1:** Timeline of symptoms and laboratory values.

Date	Pembrolizumab status	Clinical status	TSH (mU/L)	fT4/fT3	Na (mmol/L)	Levothyroxine	Notes
04 Jul 2025	Start palliative pembrolizumab	No psychiatric history; independent living	1.99	not available	135	none	Baseline TSH normal; cortisol 269 nmol/L
08 Sep 2025	Ongoing	cMRI after transient speech/tongue-control symptoms; no acute lesion	41.20	not available	130	none	Interval TSH elevation documented
~mid-Sep 2025	Ongoing	Progressive confusion, social withdrawal, functional decline	not available	not available	not available	none	Collateral history: no prior cognitive abnormalities
30 Sep 2025	Ongoing	Pre-admission laboratory evaluation	41.70	not available	124	none	Cortisol 619 nmol/L
03 Oct 2025	Paused	ED presentation: disorientation, incoherent responses, agitation	37.90	fT4 <0.5 pmol/L; fT3 <0.60 pmol/L	125; serum osm 250	Started	Urine Na 21 mmol/L; urine osm 202 mOsm/kg
04–10 Oct 2025	Paused	Fluctuating delirium; hallucinations, delusions, aggression, nocturnal agitation	not available	not available	126-131; 130 on 10 Oct	50 micrograms/day	IV midazolam required;
12–15 Oct 2025	Paused	Altered consciousness, visual hallucinations; one-to-one support	41.40 on 15 Oct	not available	129	50 micrograms/day	transfer to psychiatry
22–23 Oct 2025	Paused	Improving but residual cognitive deficits/perseveration	30.43	not available	133	Increased to 100 micrograms/day	MRI without acute explanatory lesion
30 Oct-03 Nov 2025	Paused	No longer hallucinating; residual cognitive deficits	26.16 on 03 Nov	fT4 11.3; fT3 3.0	139	100 micrograms/day	Sodium normalized before full TSH normalization
06–12 Nov 2025	Paused	Calm, coherent, oriented; no hallucinations/delusions; discharged home	15.02 on 07 Nov	fT4 12.5; fT3 3.3	136	Increased to 150 micrograms/day	
Dec 2025	Rechallenge planned	No reported neuropsychiatric recurrence	0.51	not available	137	150 micrograms/day	Thyroid replacement continued
Apr 2026	Restarted q3 weeks	Independent living; no delirium/psychosis recurrence; stable disease	0.09	not available	133	150 micrograms/day	Regular laboratory monitoring every 6 weeks

## 3 Discussion

This case describes hyperactive delirium with prominent psychotic symptoms associated with severe hypothyroidism and hypoosmolar hyponatremia during pembrolizumab therapy. The patient developed hyperactive delirium with prominent psychotic symptoms, including agitation, vivid visual hallucinations, and persecutory delusions, approximately three months after initiation of pembrolizumab. The complete remission of neuropsychiatric symptoms after levothyroxine replacement, together with the absence of previous psychiatric illness and the lack of an acute explanatory lesion on neuroimaging, supports severe hypothyroidism as the principal contributor to the clinical presentation. However, given the concomitant hyponatremia and incomplete neurological evaluation, causality cannot be established definitively.

Thyroid dysfunction is among the most frequent endocrine immune-related adverse events during immune checkpoint inhibitor therapy, particularly with programmed cell death protein 1 inhibitors such as pembrolizumab (1). Antibody negativity does not exclude ICI-associated thyroid dysfunction (2). Most cases are detected through routine laboratory monitoring or present with nonspecific systemic symptoms (2, 3, 5). In contrast, severe hypothyroidism may rarely present with acute neuropsychiatric manifestations, including delirium, hallucinations, and psychosis (4, 6). In the present case, the temporal sequence was clinically suggestive: thyroid function was normal before pembrolizumab initiation, severe hypothyroidism was documented at the time of acute neuropsychiatric deterioration, and symptoms resolved after thyroid hormone replacement and correction of metabolic abnormalities.

The diagnostic assessment also illustrates the complexity of acute mental status changes in patients receiving immune checkpoint inhibitors. Relevant differential diagnoses included immune checkpoint inhibitor-associated encephalitis (7), structural brain pathology, adrenal insufficiency or hypophysitis, metabolic encephalopathy, infection, and primary psychiatric disorder. Neuroimaging did not demonstrate an acute explanatory lesion, and normal cortisol and adrenocorticotropic hormone levels argued against adrenal insufficiency or hypophysitis. However, lumbar puncture and electroencephalography could not be performed because of severe agitation, which limits diagnostic certainty. Moderate hypoosmolar hyponatremia was also present and may have contributed to the delirium. Nevertheless, the profound hypothyroidism and subsequent complete clinical recovery after levothyroxine make pembrolizumab-associated severe hypothyroidism the most plausible primary driver. The diagnosis of hyperactive delirium was supported by altered consciousness, disorientation, incoherent thought and speech, psychomotor agitation, sleep-wake disturbance, hallucinations and delusions, and a fluctuating course. This presentation overlaps phenomenologically with myxedema psychosis; however, the prominent disturbance of consciousness and orientation, fluctuating cognitive status, motor agitation, and medical precipitants favored delirium with psychotic symptoms rather than a primary psychotic syndrome.

This case has practical implications for both oncology and psychiatry. New-onset agitation, hallucinations, psychotic symptoms, or delirium in patients receiving immune checkpoint inhibitors should prompt rapid assessment for potentially reversible immune-related endocrine toxicities, including thyroid dysfunction and adrenal insufficiency before the symptoms are attributed to a primary psychiatric disorder. This is particularly important because such presentations may initially resemble primary psychiatric illness, especially when psychotic symptoms are prominent. Early recognition and treatment may avoid prolonged morbidity, unnecessary psychiatric treatment escalation, and premature discontinuation of effective oncological therapy.

Another relevant aspect of this case is the successful rechallenge with pembrolizumab. After clinical stabilization and adequate thyroid hormone replacement, pembrolizumab was restarted at three-week intervals with close laboratory monitoring and without recurrence of delirium or psychotic symptoms. Although conclusions from a single case must remain cautious, this observation suggests that rechallenge may be feasible after stabilization of endocrine immune-related adverse events, provided that careful interdisciplinary follow-up and regular thyroid monitoring are ensured.

The main limitation of this report is that causality cannot be proven definitively. The clinical presentation was probably multifactorial, with hyponatremia, sleep disruption, psychosocial stress, and possible medication-related factors potentially contributing to the delirium. No structured delirium scale was applied, although the clinical documentation supported the diagnosis. In addition, the absence of lumbar puncture, electroencephalography and serum antineuronal or onconeural antibody testing means that immune-mediated encephalitis or subclinical epileptic activity could not be fully excluded. A further limitation is that thyroid antibodies were not measured during the acute episode. Delayed testing in August 2026 showed mildly elevated thyroperoxidase antibodies with negative TSH receptor and thyroglobulin antibodies. These results suggest thyroid autoimmunity but cannot determine whether antibodies were already present at the onset of thyroid dysfunction or emerged later during the course of immune checkpoint inhibitor therapy. In addition, the thyroid assay platform, ACTH values, and a comprehensive assessment of other pituitary axes were not available in the records reviewed. Finally, as a single case report, the findings are not generalizable. Despite these limitations, the case highlights an important and reversible cause of acute neuropsychiatric deterioration during pembrolizumab therapy and supports systematic endocrine evaluation in similar clinical scenarios.

## 4 Patient perspective

The patient reports good tolerance of the ongoing pembrolizumab therapy, which is currently administered every three weeks. Following the temporary interruption during hospitalization, restaging demonstrated stable disease. With regular follow-up, including laboratory monitoring every six weeks (including TSH and cortisol), she feels clinically stable and expresses satisfaction with her present condition and the ongoing treatment.

## Data Availability

The original contributions presented in the study are included in the article/supplementary material. Further inquiries can be directed to the corresponding author.
